# Wound healing after therapy of oral potentially malignant disorders with a 445-nm semiconductor laser: a randomized clinical trial

**DOI:** 10.1007/s00784-023-05438-9

**Published:** 2023-12-26

**Authors:** Axel Meisgeier, Paul Heymann, Thomas Ziebart, Andreas Braun, Andreas Neff

**Affiliations:** 1 Department of Oral and Craniomaxillofacial Surgery, UKGM GmbH, University Hospital Marburg, Giessen/Marburg, Germany; 2 Faculty of Medicine, Philipps-University, Marburg, 35043 Marburg, Germany; 3 Department of Operative Dentistry, Periodontology and Preventive Dentistry, RWTH University Aachen, Aachen, Germany

**Keywords:** Diode laser, Leukoplakia, OPMD, Oral precursor lesion, Oral squamous cell carcinoma, OSCC, Oral surgery

## Abstract

**Objectives:**

Oral potentially malignant disorders (OPMDs) are the most clinically relevant precursor lesions of the oral squamous cell carcinoma (OSCC). OSCC is one of the 15 most common cancers worldwide. OSCC is with its high rate of mortality an important cause of death worldwide. The diagnosis and therapy of clinically relevant precursor lesions of the OSCC is one of the main parts of prevention of this malignant disease. Targeted therapy is one of the main challenges concerning an oncologically safe tissue removal without overwhelming functional and aesthetic impairment.

**Materials and methods:**

In this randomized controlled trial, a newly introduced intraoral 445-nm semiconductor laser (2W; cw-mode; SIROLaser Blue, Dentsply Sirona, Bensheim, Germany) was used in the therapy of OPMDs. Duration and course of wound healing, pain, and scar tissue formation were compared to classical cold blade removal with primary suture by measuring remaining wound area, tissue colorimetry, and visual analogue scale. The study includes 40 patients randomized using a random spreadsheet sequence in two groups (*n*1 = 20; *n*2 = 20).

**Results:**

This comparative analysis revealed a significantly reduced remaining wound area after 1, 2, and 4 weeks in the laser group compared to the cold blade group (*p* < 0.05). In the laser group, a significantly reduced postoperative pain after 1 week was measured (*p* < 0.05).

**Conclusion:**

Laser coagulation of OPMDs with the investigated 445-nm semiconductor laser is a safe, gentle, and predictable surgical procedure with beneficial wound healing and reduced postoperative discomfort.

**Clinical relevance:**

Compared to the more invasive and bloody cold blade removal with scalpel, the 445-nm semiconductor laser could be a new functional less traumatic tool in the therapy of OPMDs. The method should be further investigated with regard to the identification of further possible indications.

**Trail registration:**

German Clinical Trials Register No: DRKS00032626.

## Introduction

Oral potentially malignant disorders (OPMDs) are the most clinically relevant precursor lesions of the oral squamous cell carcinoma (OSCC). OSCC is one of the 15 most common cancers worldwide [1]. Two-thirds of all patients are men with a mean age at first diagnosis of 62 years, while women are diagnosed with a mean age of 66 years [2]. Women show an overall 5-year survival rate of 61% compared to 48% in men. The reason for that is mainly the higher rate of alcohol and tobacco consumption in men and the diagnosis at an earlier tumor stage in women. So one of three tumors is diagnosed at T1 stage in women but only one of four in men [2–5]. Despite aggressive multimodal therapy options, the 5-year survival rate has improved only insignificantly over the past four decades. The prognosis of oral cancer is mainly determined by the stage of the tumor and the nodal status at the time of diagnosis. If oral squamous cell carcinoma is diagnosed and treated at an early stage, the disease-free survival rate is significantly more favorable compared to the late diagnosis. The therapy-associated functional limitations are also significantly lower, and the post-therapeutic quality of life is better [5].

The diagnosis and therapy of clinically relevant precursor lesions of the oral squamous cell carcinoma is one of the main parts of prevention of this malignant disease. Targeted therapy is one of the main challenges concerning an oncologically safe tissue removal without overwhelming functional and aesthetic impairment. Compared to the more invasive and bloody cold blade removal with scalpel, the 445-nm semiconductor laser could be a new functional less traumatic tool in the therapy of OPMDs.

Laser devices of a respective wavelength are able to cut or ablate tissue with synchronous hemostasis. Many interaction effects with the tissue have been described such as thermal, photochemical, or ablative effects [6].

Surgical devices with CO2 and Nd:YAG (neodymium-doped yttrium aluminum garnet) lasers have already been described for soft tissue dental surgery procedures [7]. Short-wave lasers have been described in several medical fields with the argon lasers in dermatology or ophthalmology where the use was limited by the high effort in inducing and controlling a surgical usable laser beam [8, 9]. With the evolving progress in optoelectronics, semiconductor lasers offer an increasing number of applications in research, industry, and medicine due to their compact structure, high optoelectronic efficiency, and reliability.

For oral applications recently, a 445-nm semiconductor laser device was introduced [10, 11]. One of the main benefits of the 445-nm blue laser compared to the standard red laser systems is its shorter wavelength, and the higher absorption in the target tissue leads to less deep penetration and less backscatter [10].

The reduced energy absorption in surrounding tissues from scattering may lead to less thermal side effects and promote better wound healing. Thus, the 445-nm laser technology should provide an auspicious instrument for a variety of applications such as surgical incision and ablative tissue surgery. Blue laser may offer a bloodless and clean cutting option with only few side effects in adjacent tissues basing on its special absorption properties [12].

However, there is no study that analyzed the effects of ablative tissue surgery in a randomized controlled trial. Furthermore, there is no extensive information about blue light laser cutting properties so far. Therefore, the aim of the present study was to establish and evaluate the short-term effects of laser coagulation of OPMDs with a novel 445-nm semiconductor laser, examining the assumption of a blue light laser being more effective and comfortable than conventional cold steel excision without increasing side effects.

## Materials and methods

### Patients

This investigation was designed as a controlled randomized clinical trial. The study protocol was approved by the Institutional Ethics Board at Philipps-University Marburg, Germany (133/17; November 7, 2017). Planning and execution followed the rules in respect of the declaration of Helsinki. Informed consent was obtained from each patient after a detailed explanation of the nature, risks, and benefits of the clinical procedures to be performed. The study population comprised 40 patients with oral potentially malignant disorders (OPMDs) diagnosed by small incisional biopsy prior to inclusion to the study. The subjects were included from January 2020 to July 2020 according to the eligibility criteria. This trial was registered at German Clinical Trials Register (https://www.drks.de) No: DRKS00032626.

### Inclusion criteria

Patients were included when they were older than 18 years and had a diagnosed OPMD in need of treatment matching to have no dysplasia, mild dysplasia, or moderate dysplasia. All subjects signed the formal consent to participate in the study after receiving an explanation of risks and benefits from an individual who was not a member of the present study.

### Exclusion criteria

Patients with systemic contraindications against surgical procedures, those under conditions or medication that would interfere with the wound healing, or patients with diagnosed severe dysplasia were excluded.

### Treatment

Patients were randomly allocated in one of the following groups:

- Group 1 (test, *n* = 20): ablation of the OPMD with a 445-nm semiconductor laser (SIROLaser Blue, Dentsply Sirona, Bensheim, Germany)
- Group 2 (control, *n* = 20): cold blade removal of the OPMD

Randomization was done using a random spreadsheet sequence to allocate patients in each group by one individual who was not a member of the study (a department secretary).

### Surgical procedure

All subjects were instructed about the causes and consequences of OPMDs including risk factors in connection with the origin of OPMDs, such as alcohol and tobacco consumption, mechanical irritation, or chronic inflammation. Before the surgical procedure, operation areas were disinfected using 0.2% chlorhexidine solution (Chlorhexamed Forte 0.2%, GlaxoSmithKline GmbH, Munich, Germany). Local anesthesia was performed using articaine with adrenalin (Ultracain D-S 1:200,000, Sanofi-Aventis GmbH, Frankfurt, Germany). The surgical procedure was performed by one surgeon (AM). In group 1, the OPMD was destructively ablated to its clinical borders with a narrow seam of clinically healthy tissue using the 445-nm semiconductor laser in continuous wave mode with a power setting 2.0 W in slight non-contact mode until carbonization was seen. The device setting of 2.0 W corresponded to an effective power of 1.72 W at an average working tip angle of 45°. Assuming a Gaussian laser beam profile, the power density was 4280 W/cm^2^ (fiber tip diameter 0.32 mm, 1.72-W output power). No suture or wound dressing was applied. In group 2, the excision was performed with a N° 15 single-use blade (Feather Co. Ltd., Osaka, Japan) to its clinical borders with a narrow seam of clinically healthy tissue.

### Postoperative care

All patients were instructed to use 400 mg of ibuprofen every 8 h for 3 days in case of postoperative pain. Sutures were removed after 7 days before further photo documentation.

### Evaluation parameters

The following measures were recorded:

1. Remaining wound area (RWA): Defect area was measured straight after operation and after 7, 14, and 28 postoperative days. For this, photographs standardized for brightness, distance, and angle were taken (Nikon D3500, Nikon Inc., Minato, Japan). A scale was used as a reference to measure this area. These photographs were exported to an image software (ImageJ—NIH, Bethesda, USA), and the wound area was measured in square millimeters [13].
2. Tissue colorimetry (TC): Tissue color similarity of regions adjacent to the operated area was analyzed through photographs. The photographs were exported to an image software (ImageJ—NIH, Bethesda, USA), and two areas, one from the wound and another adjacent, were used. Color difference (ΔE) of the areas was evaluated using brightness parameters (L), red-green chroma scale (a), and yellow-blue chroma scale (b) according to the following equation [14]:
  $$\Delta E={({({L}_{wound}-{L}_{adjecent})}^{2}+{({a}_{wound}-{a}_{adjacent})}^{2}+{({b}_{wound}-{b}_{adjacent})}^{2})}^{(\frac{1}{2})}$$
3. Postoperative discomfort (D): Through air spray for 5 s over the operated site, sensitive function was measured at 7, 14, and 28 days after surgical procedure. After air spray application, patients were required to use a visual analogue scale (VAS) of 100 mm to assess discomfort; scale extremes were “no pain” to “extreme.” In addition, patients were asked to report the number of analgesics taken during the first week [13].

### Examiner calibration

One examiner was responsible for performing clinical parameters and photograph measurements (AM). The examiner underwent a calibration training for area measurements. Ten photographs of subjects who were not participating in the study were taken. An intraclass correlation test was performed. The examiner was trained until the agreement reached 90% for the area measurements.

### Data analysis

During the descriptive phase, data were expressed as mean ± standard deviation or expressed in percentages. Data were analyzed according to distribution by the Kolmogorov–Smirnov test. Unpaired *t*-test was used for intergroup comparison of the remaining wound area, tissue colorimetry analysis, postoperative discomfort parameter, and operation time. For all tests, the significance level of 5% was used. Statistical analysis was performed using IBM SPSS Statistics Version 24.0 (SPSS GmbH, Munich, Germany).

## Results

**Fig. 1:**
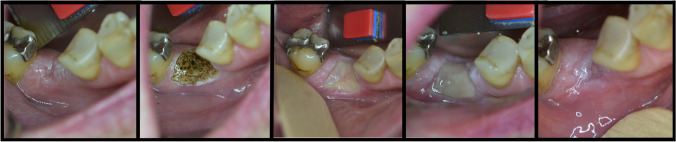
Clinical view of the wound healing after 445-nm laser ablation: **a** before surgery; **b** postoperative view; **c** 7 days follow-up; **d** 14 days follow-up; **e** 28 days follow-up

**Fig. 2:**
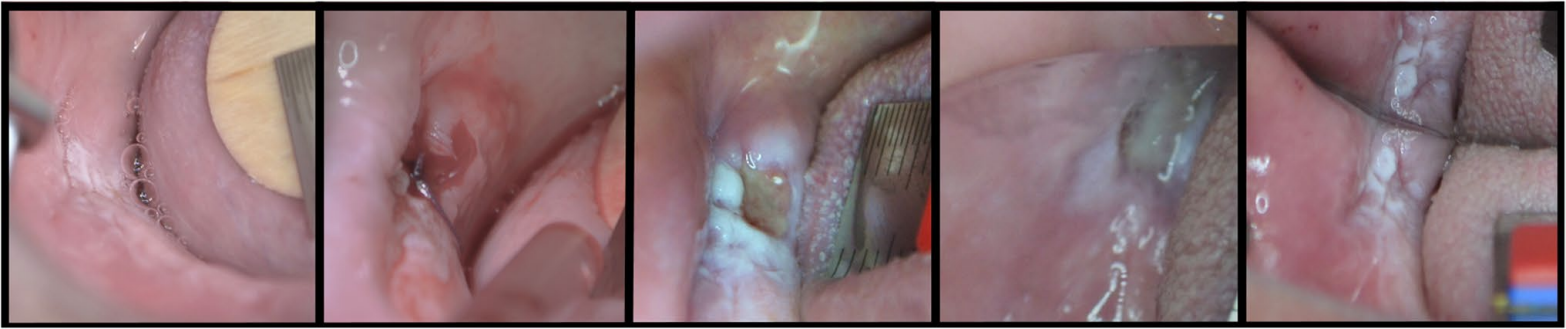
Clinical view of the wound healing after cold blade excision: **a** before surgery; **b** postoperative view; **c** 7 days follow-up; **d** 14 days follow-up; **e** 28 days follow-up

Forty patients were randomized for one of the two groups. None of them presented adverse effects to the procedures or the laser application protocol. Table 1 shows the demographic characteristics of the patients. There were no significant differences in clinical parameters between the two groups at baseline. Figures 1 and 2 show representative photo documentation at the points in time examined in both groups.

**Table 1:** Patient characteristics of the OPMD patients included in the analysis (*n* = 40)

	Group 1: 445-nm laser (*n* = 20)	Group 2: excision (*n* = 20)
Gender		
Male	8	12
Female	12	8
Age	62.50 ± 15,63	63.00 ± 16,92
Localization		
Alveolar crest	11	8
Buccal plane	4	7
Tongue	3	3
Floor of the mouth	1	1
Palate	1	1
Dysplasia		
No dysplasia	18	19
Mild dysplasia	2	1
Moderate dysplasia	0	0
Side		
Right	9	9
Left	10	10
Both	1	1
Jaw		
Maxilla	2	2
Mandible	11	8
Undefined	7	10

### Remaining wound area (RWA)

**Fig. 3:**
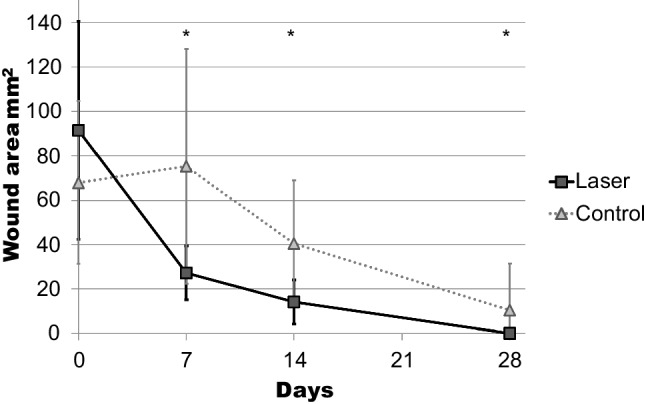
Wound area (in mm.^2^) of the two groups. Statistically significant differences were found 7, 14, and 28 days after surgery (*p* < 0.05; *)

The measurements of the remaining wound area presented a significant reduction during the observation period for both groups (Fig. 3). An intragroup comparison showed that the wound areas at day 7 were statistically greater than they were at day 14, and these were statistically greater than at day 28. At day 7, measurements were 27.3 ± 12.1 mm^2^ for the test group and 75.2 ± 52.9 mm^2^ for the control group. At day 14, measurements were 14.1 ± 10.1 mm^2^ for the test group and 40.6 ± 28.6 mm^2^ for the control group. At day 28, measurements were 0.04 ± 0.19 mm^2^ for the test group and 10.5 ± 21.0 mm^2^ for the control group. A statistically significant difference was observed between the two groups at all follow-up appointments; the group that received the laser presented significantly smaller wounds.

### Tissue colorimetry (TC)

**Fig. 4:**
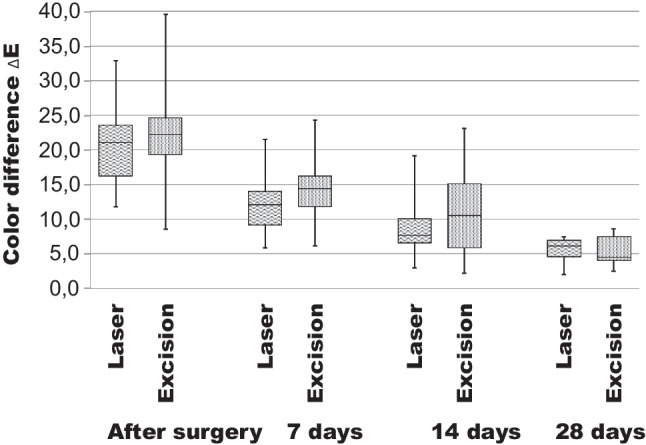
Tissue colorimetry (TC): Color difference (ΔE) of the two groups. There were no statistically significant differences found after surgery and after 7, 14, or 28 days (*p* < 0.05)

Colorimetry analysis revealed that both groups presented similar color patterns (Table 2). Both groups showed continuous reduction of color difference over time (Fig. 4). None of the patients presented clinical notable scar formation in the follow-up period. There were no statistically different color patterns between the two groups at any time (*p* < 0.05).

**Table 2:** Parameters at baseline and after 7, 14, and 28 days

		Baseline	7 days	14 days	28 days
TC (ΔE)	Test	21.1 ± 5.8	12.1 ± 4.1	7.7 ± 3.9	6.1 ± 2.4
	Control	24.6 ± 9.0	16.2 ± 5.8	10.5 ± 7.7	4.5 ± 2.4
T (min)	Test	10.2 ± 5.6	-	-	-
	Control	7.4 ± 4.3	-	-	-
D (VAS)	Test	-	1.6 ± 1.3	0 ± 0	0 ± 0
	Control	-	3.7 ± 2.8	0.2 ± 0.2	0 ± 0

*TC* tissue colorimetry, *T* operation time in minutes, *D* postoperative discomfort

### Operation time (T)

There were only small differences between the operation time in both groups. While the laser ablation was measured with 10.2 ± 5.6 min, the conventional cold blade excision took 7.4 ± 4.3 min. There was no significant difference (*p* < 0.05).

### Postoperative discomfort (D)

Patients generally reported mild discomfort after both laser ablation and cold blade excision. At day 7, the average VAS for discomfort was 1.6 ± 1.3 for the test group and 3.7 ± 2.8 for the control group which is a statistically significant difference between the groups (*p* < 0.05). After 14 days, discomfort was reduced to 0.0 ± 0.0 for the test group and 0.2 ± 0.2 for the control group with no statistically significant difference between groups. At day 28, neither group presented discomfort.

## Discussion

Cold blade excision is the gold standard in the therapy of oral potentially malignant disorders but may lead to postoperative patient discomfort or functional impairing. Scar tissue formation after resection of the lesions may require surgical reconstruction. Conventional laser systems as CO2 lasers are technically sophisticated, are expensive, and are not feasible in many outpatient settings. Thus, an interesting alternative would be to minimize the negative impact of the cold blade excision without losing the positive therapeutic benefits. The aim of this study was the first evaluation of a recently established compact 445-nm semiconductor laser in this specific indication considering wound healing and patient discomfort in comparison to the standard cold blade excision.

The present study showed that wound healing after laser ablation with the 445-nm laser is favorable compared to the standard therapy. Remaining wound area after laser therapy shows a predictable continuous decrease over time with significantly smaller remaining wound areas in the laser group. No relevant wound area was measured after 4 weeks in the laser group. Tissue colorimetry showed comparable wound healing after laser and excision therapy without significant differences. This implicates a comparable predictability with a little shorter wound healing course in the laser group. There is a lot of literature supporting a wound healing propagation by laser light. Laser light of different wavelengths was shown to support wound healing in different intraoral an extraoral settings. Parker et al. describe in a recent systematic review on different short- and long-wave laser wavelengths for intraoral indications, positive effects of laser light on wound healing compared to scalpel treatment in 46%, and no difference in 46% of the included studies [15]. However, none of the included studies evaluated wound healing after the use of short-wave lasers (< 650 nm) comparing to a non-laser comparison group at a clinical level. Gobbo et al. describe excellent wound healing after excision of benign oral lesions with blue diode laser in comparison to infrared diode laser or quantic molecular resonance scalpel [16]. Dias et al. describe a positive effect of laser light on oral wound healing of free gingival and connective tissue grafts using a 660-nm diode laser [13]. Dawood et al. show a positive effect of short- and middle-wave laser light in a mouse model [17]. Fahimipour et al. also show a positive effect of laser light on oral wound healing in a mouse model [18]. Serrage et al. discuss different mechanisms of photobiomodulation by blue laser light promoting wound healing [19]. The modulation of the production of reactive oxygen species (ROS) is probably a central point. This is influenced by various signaling pathways of inflammation and regeneration, which include interleukin 6 (IL-6), transforming growth factor beta (TGF-β), and fibroblast growth factor 2 (FGF-2). Noda et al. showed the induction of osteocalcin gene in dental extraction sockets in rats leading to a significantly higher number of dividing cells in the early phase of wound healing and subsequently a higher bone density and bone volume after final wound healing using a 910-nm diode laser [20]. A widely known and published effect contributing to intraoral wound healing may the antibacterial effect of short-wave laser light [21, 22]. Lipovsky et al. show an antibacterial effect of short-wave laser light in terms of promoting and accelerating wound healing [23]. Further scientific studies are required to investigate other possible wavelength-specific effects.

After 1 week, the postoperative pain perception in the laser group was significantly lower than in the comparison group. Even if this can only be an indication in the context of an unblinded study and without recording the actual use of analgetics in the outpatient setting and its influence on the assessment of the pain intensity, this implies a possible analgesic effect of the laser treatment compared to the classical surgical approach. The scientific data on this is promising. Lopez-Jornet et al. were able to show reduced pain perception after treatment of oral leukoplakia using a CO2 laser in comparison to classic excision using a scalpel in a randomized controlled study [24]. Rocca et al. describe a positive effect of short-wave laser therapy on pain relief in oral aphthae treatment [25]. Likewise, Suter et al. describe a pain-relieving effect of laser therapy in oral aphthae [26]. Such effects could not only be described in the field of intraoral soft tissue surgery. In the field of endodontics too, there is a scientific consensus on the reduction of post-endodontic complaints through laser-assisted root canal decontamination [27–29]. In the field of wisdom tooth surgery, there are indications of possible postoperative pain reduction through laser-assisted osteotomy and through postoperative low-level laser therapy [30, 31]. In addition, low-level laser therapy appears to have an overall positive effect on postoperative pain perception. Thus, D'Avila et al., for example, even demonstrate a positive effect of this treatment after bimaxillary osteotomy [32]. In addition, there are numbers of studies from all parts of the medical spectrum that show a positive effect of laser radiation, for example, on neuropathic pain in the context of diabetic foot syndrome [33, 34]. Cronshaw et al. discussed a photobiomodulation effect involving transmembrane uncoupling proteins promoting cellular heat stress response as a key factor in laser associated analgesia [35]. With regard to other physiological signal cascades involved, animal experiments have shown a possible influence on the endogenous opioid system and an associated reduced perception of pain after laser treatment [36, 37]. To date, however, there is no scientific consensus on the specific influence of different wavelengths, in particular on the short-wave lasers investigated here, although a possible wavelength-specific effect seems likely [38]. Further investigations, in particular double-blind randomized controlled clinical trials, are necessary.

Various wavelengths and protocols for the use of diodes, gas, and solid-state lasers have been published, but there is no uniform consensus regarding the optimal procedure, wavelength, and the time course of the energy deposition [39]. The current, widely recognized therapy standard in laser therapy for potentially malignant lesions of the oral mucosa is the use of CO2 lasers. With their laser wavelength of around 10,000 nm, these lasers are mainly absorbed in water, so that the tissue interaction is mainly vaporizing-thermal. Today, lasers that work in this way usually require an optical power of around 1–3 W. By using a short-wave diode laser around 450 nm in the blue visible spectrum whose absorption maxima are in the range of hemoglobin and melanin, this required optical output power can be reduced with the same cutting performance as systems operating with longer wavelengths [10, 40]. This may allow slimmer designs of the overall systems and of the intraoral applicators improving the handling and the clinical efficiency of the application in particular when treating awake patients who can only be positioned freely to a limited extent. The low laser wavelength with its absorption maximum in the area of hemoglobin and melanin can penetrate deeper into the tissue compared to the CO2 laser mainly absorbed by water [41]. It remains unclear if this can reduce the overall tissue load, particularly that of the surrounding tissue. Deeper penetration may lead to a potential therapeutic effect on deep-seated cells which could be desirable in the context of addressing possibly recurrent benign lesions of the oral mucosa and should be checked in further studies.

The duration of the intervention differed only slightly in both patient groups with only a minor, non-significant tendency to prolong the operating time through the use of laser coagulation. The laser coagulation of potentially malignant lesions of the oral mucosa using a 445-nm semiconductor laser, like the laser-based excision methods already described extensively in the literature, represents a time- and resource-efficient therapy method that can be easily integrated into the clinical process with little additional effort [42–44].

An effective therapy of potentially malignant lesions of the oral mucosa was achieved according to clinical standards using the recently established 445-nm semiconductor laser. This study is subject to certain limitations, which are mainly related to the population available for analysis. First, there is a selection bias regarding patients suffering from OPMDs, as potential risk factors such as tobacco use or chronic mechanical irritation may also affect wound healing differently in the laser and excision group compared to the general population. Second the limited number of participants (*n* = 40) does not allow significant interpretation of small effects especially in regard of the used tissue colorimetry. This would, however, be interesting to evaluate further in detail in future studies. In addition, the questions of direct comparison to CO2 laser systems regarding wound healing and postoperative pain perception as well as the long-term temporal and quantitative evaluation of recurrences in comparison to established standard methods remain groundbreaking for future use in this indication. The molecular-biological basis of the influence of short-wave laser light on wound healing and pain perception represents a wide field for future scientific investigations and forms the basis of a well-founded description of clinical effects to be observed. As a further development, an extension of the mentioned indications to other indication areas would be conceivable and desirable. The used laser device was set to its maximum technical performance in this indication. For deeper resections, for example, in squamous cell carcinomas of the oral cavity, further technical considerations are required, possibly with the option of a further increase in optical and temporal performance in order to be able to compete with established systems such as the CO2 laser. Overall, the results shown demonstrate the attractiveness of short-wavelength diode lasers as an enrichment of the surgical instrument portfolio. The potential spectrum of their applicability extends far beyond intraoral use.

## Conclusions

Laser coagulation of OPMDs with the investigated 445-nm semiconductor laser is a safe, gentle, and predictable surgical procedure. The method should be further investigated with regard to the identification of further possible indications.

## Data Availability

The data presented in this study are available on request from the corresponding author.
